# Synthesis of Jicama (*Pachyrhizus erosus*) Starch Particles by Electrospraying: Effect of the Hydrolysis Degree

**DOI:** 10.3390/polym17152069

**Published:** 2025-07-29

**Authors:** Fatima Sarahi Serrano-Villa, Eduardo Morales-Sánchez, José Alfredo Téllez-Morales, Verónica Cuellar-Sánchez, Reynold R. Farrera-Rebollo, Georgina Calderón-Domínguez

**Affiliations:** 1 Departamento de Ingeniería Bioquímica, Escuela Nacional de Ciencias Biológicas, Instituto Politécnico Nacional, Ciudad de México 07738, Mexico; fserranov2001@alumno.ipn.mx (F.S.S.-V.);; 2 Centro de Investigación en Ciencia Aplicada y Tecnología Avanzada, Instituto Politécnico Nacional, Querétaro 76090, Mexico; emoraless@ipn.mx

**Keywords:** electrospraying, jicama starch, acid hydrolysis, particle synthesis, thermal, rheological and structural starch properties

## Abstract

Electrohydrodynamic atomization (EHDA) has significant advantages for microencapsulating compounds in various structures using biopolymers, where more research using pure starch is required. Concerning this, jicama starch and its hydrolysates have not yet been tested, despite their unique characteristics, which come from an alternative low-value-added crop source. Rapid acid hydrolysis of jicama starch with H_2_SO_4_ resulted in dextrins with a degree of hydrolysis (DE) from 0.4 to 19% within 1–12 h, and syrup solids at 24 h (DE = 42%). This process modifies the water retention capacity of jicama starch, gel viscosity, surface tension, and electrical conductivity. Hydrolyzed starch particles obtained by electrospraying (10 kV, L = 10 cm, Q = 2 mL/h) showed Feret diameters and roundness significantly influenced (*p* ≤ 0.05) by the degree of hydrolysis rather than the concentration of solids. It was found that hydrolyzed jicama starch with a DE < 6.3% can be used as the sole wall material to form particles by electrospraying, as they facilitate the formation of stable and rounded like-microspheres particles; this was not feasible above this threshold. The results suggest that the jicama starch’s ability to be used as a wall material in the electrospray synthesis of particles or microspheres appears to be determined by the degree of hydrolysis.

## 1. Introduction

In the food industry, it is common practice to include functional compounds in formulations, looking to develop foods with different technological or nutraceutical properties. For example, probiotics, as well as other compounds with nutritional, therapeutic, and protective purposes, or those that improve food sensory desirable characteristics such as texture, flavor, aroma, and color, among others, are commonly included [1]. In this regard, microencapsulation (including microspheres and microcapsules) is commonly used to fulfill this purpose [2,3].

There are different techniques for preparing microcapsules, including electrohydrodynamic atomization (EHDA), spray drying, extrusion, freeze drying, ultra-high pressure, coagulation, fluidized bed coating, molecular inclusion, chemical, and enzymatic methods [4]. These techniques have disadvantages, such as the use of high temperatures and/or the need for more complex equipment and materials [5].

Compared to other techniques, EHDA, also known as electrospraying, has significant advantages such as ease of operation, low cost, straightforward structural adjustments, and the ability to incorporate nutrients and complex molecules easily [6] in the absence of heat treatment, gaining special importance in the food and pharmaceutical areas during the last decade [7,8].

The process’s behavior and the electrospraying product’s final morphology are influenced by the interaction of Coulombic, inertial, electric field, viscoelastic, and surface tension forces [9]. Consequently, electrohydrodynamic atomization can generate different structures based on process conditions (applied voltage, flow rate, distance to the collector, equipment configuration), the properties of the raw materials used in the polymeric solution including the type, structure, and concentration of the polymer, as well as electrical conductivity, surface tension, and viscosity, among others [10,11]. Cuellar-Sánchez et al. [9] cited that EHDA products can be synthesized with raw materials from different origins, among which proteins (gelatin, pectin, zein) and polysaccharides (chitosan, alginate, starch) are widely used.

Starch is a biopolymer formed by α-D-glucose units linked by α (1 → 4) and α (1 → 6) glycosidic bonds, arranged in two types of high molecular weight molecules: amylose and amylopectin [12]. The internal structure of starch granules is described by the growth rings model, which is composed of alternating semi-crystalline (amylopectin) and amorphous (amylose) regions [13]. Starch is used as a standard wall material in the food industry to produce delivery systems for functional compounds, nutrition enhancers, and food preservatives, among others [4]. Starch, as wall material for encapsulation technology, has advantages such as being biocompatible, stable, widely available, low cost, tasteless, completely biodegradable, and can be modified into derivatives with different desired properties by functionalization or alteration of its granular structure [5].

The methods used to modify the physicochemical and functional properties of starch include physical, biochemical, genetic, and chemical mechanisms or a combination of them [14]. Among these methods, starch gelatinization is a physical modification that allows the creation of an encapsulating material, which envelopes the compound of interest when starch gels retrograde [15]. On the other hand, acid hydrolysis is a chemical modification and the simplest and most common method applied to modify starch [16,17]. Acid hydrolysis produces different starch structures and derivatives as a function of the process conditions applied, which include temperature, hydrolysis duration, acid concentration, type of acid, agitation speed, and starch structural characteristics [18].

Starch treatment with hydrochloric or sulfuric acid has been shown to cause depolymerization of the amylose and amylopectin chains [19,20]. Thus, dextrins, maltodextrins, solid syrups [21], and nanocrystals are produced by varying parameters such as hydrolysis time, acid type, and concentration, among others [15,22]. Consequently, hydrolyzed starch exhibits an increase in the enthalpy change (ΔH), as well as in the temperature rank of gelatinization endotherms (or the peak gelatinization temperature). This treatment also increases starch solubility and crystallinity, swelling power, and a loss of pasting viscosity [13,17].

Starch is commonly extracted from vegetables, with maize and potatoes serving as the primary sources for industrial use, and at a lower proportion, tuberous roots [9,12]. Over the past decade, the interest in utilizing non-conventional sources to produce starch has increased, particularly agro-industrial residues or low-added-value crops that do not compete as a crucial food source for human populations and exhibit different properties [23]. In this context, jicama root (*Pachyrhizus erosus*) is an example. It contains significant amounts of starch (40–60% on a dry basis) at commercial maturity [24].

Jicama starch presents polygonal, spherical, and irregular granules with a smooth surface, ranging in size from 3 to 21 µm [25]. Its structural arrangement is predominantly of the CA type polymorphism, with a relative crystallinity of 29 to 44%. Regarding its chemical composition, it has been reported to present a variable content of amylose (11–25%) depending on the genetic variety [23] and less than 1% of proteins, lipids, and ashes, in which calcium, potassium, and phosphate are higher than in potato starch [24,25].

To our knowledge, jicama starch has not been tested in the electrohydrodynamic technology. As a wall material of importance in the electrospraying process, starch is commonly used as a modified compound. Cuellar-Sánchez et al. (2022) [9] cited in this regard that gelatinized and chemically modified starches are a very common material for capsule and fiber synthesis. However, studies using starch as the only polymer are limited and require further investigation in the food field [26]. Research on the formation of starch-based capsules and particles from electrohydrodynamic spraying techniques has focused on the use of modified maize starch, such as resistant [27,28,29] debranched [30], resistant maltodextrins [31,32], and octenyl succinated starch [33]. The studies often involve the use of copolymers, including gums [31], chitosan, and alginate [29], among others, and surfactants like Tween 80 [32], Span 20, and lecithin [31] to enhance the solution properties to be electrosprayed.

Physical properties of the starch solutions to be electrosprayed, such as viscosity, polymer concentration, and molecular weight of the polymer, play important roles [9]. For example, in some studies, solutions based on potato starch solubilized in formic acid were processed to enhance electrospinning fiber production [34,35]. The authors attribute this to starch formylation, its chemical gelatinization, and a reduction in its molecular weight through hydrolysis, which also modifies the viscosity and influences the role of surface tension.

We hypothesize that the properties of starch solutions can be modified by acid hydrolysis with sulfuric acid to improve the EHDA process. This acid is known to produce more specific cleavages than HCl [14,36], and it could form sulphate esters [22] that allow it to interact with compounds of food interest to be encapsulated, such as proteins or enzymes like glucose oxidase in breadmaking. The present work aims to evaluate the effect of acid hydrolysis on the preparation of microspheres from jicama (*Pachyrhizus erosus*) starch using simple electrohydrodynamic atomization.

## 2. Materials and Methods

### 2.1. Materials

Jicama tubers (*P. erosus* L. Urban, variety) from San Juan, Nayarit, Mexico, were used. All reagents were analytical grade. Sodium bisulfite (3556-20) and phenol (2058-01) were bought from JT Baker, acetone (6016) from Fermont, and sulfuric acid (EMSURE^®^, 1.00731), D-glucose (G8270), Tween 80 (10099956), and glycerol (G9012) from Sigma-Aldrich. The enzymatic kits K-TSTA-100A 02/22 and K-AMYL 06/18 were supplied by Megazyme. Starch cup (998118), SPC rotor (999188), and DIN SST (999292) geometries used in RVA analysis and flow curves were supplied by TA Instruments. Finally, distilled water was used in all experiments and was produced in a local potabilization plant.

### 2.2. Starch Extraction

The method proposed by González-Lemus et al. [25] was followed, in which the jicama tuber was rinsed and cut into cubes (1 cm^3^); it was then soaked for 30 min in a 1500 ppm solution of sodium bisulfite (3556-20, J.T. Baker, Avantor Performance Materials, Center Valley, PA, USA) in a 1:3, root-solution (*w*/*v*) ratio. The jicama cubes were placed in a plastic bag, immersed in a dry ice/acetone bath for 10 min, and stored at −24 °C for 24 h. The cubes were then thawed, defrosted, and stored in a dry ice/acetone bath. They were then thawed (12 h, 25 °C) and ground (jicama-water 1:3 *w*/*v*) in an Oster PS blender for 60 s. The resulting suspension was processed in an ultrasonic bath (Cole-Parmer 08895-69, 40 kHz, Vernon Hills, IL, USA) for 10 min. It was then filtered through a plastic mesh 250 μm (Monyl, Sefar Nytal PA, Switzerland) and centrifuged (Dynamica Velocity 18R, Metrix, UK) at 4500× *g* for 10 min at 4 °C. The supernatant was removed, and the precipitate dried at 30 °C for 24 h (AFOS Ltd., Afos Mini-Klean, Hull, UK).

### 2.3. Starch Acid Hydrolysis

The hydrolysis was carried out following the methodology outlined by López-Hernández et al. (2022) [36], with some adaptations: Starch dispersions containing 15% (*w*/*v*) of native starch were prepared in a 3.16 M sulfuric acid solution (1.00731, EMSURE, SIGMA, St. Louis, MO, USA). These dispersions were then incubated at 40 °C with constant agitation at 150 rpm in a shaking incubator (Barnstead Lab-Line A-Class, Barnstead international, Dubuque, IA, USA) between 1 and 24 h [20]. The time interval was selected based on the results from preliminary tests; times longer than 24 h produced insoluble solids, which do not allow the formation of stable solutions, making them unable to be processed by electrospraying. The samples were washed with distilled water consecutively until a pH as close to neutral as possible was achieved. In each case, the starch was recovered by centrifugation at 4500× *g* at 4 °C for 5 min (Dynamica Velocity 18R, Metrix, UK). The starch was then dried at 30 °C in a convection oven (AFOS, Hull, East Yorkshire, UK) and stored in a desiccator with anhydrous Drierite^TM^ (Drierite, Xenia, OH, USA) at room temperature for later use.

### 2.4. Hydrolysis Degree

The degree of hydrolysis was developed based on Sánchez de la Concha et al. [14], measured from the first supernatant at the end of the hydrolysis incubation, through the phenol method developed according to González-Vázquez et al. [37], where 1 mL of sample is mixed under dark conditions with 0.6 mL of phenol (2058-01, J.T. Baker, Avantor Performance Materials, Center Valley, PA, USA) at 5% (*w*/*v*), and 3.6 mL of concentrated H_2_SO_4_ (1.00731, EMSURE, SIGMA, St. Louis, MO, USA), and incubated for 30 min at 25 ± 1 °C. Subsequently, the reaction is stopped in an ice bath, and the absorbance is read at 490 nm (Multiskan Go spectrophotometer; Thermo Fisher Scientific; Vantaa, Finland). The values are quantified with a standard curve from 0 to 100 µg/mL D-glucose (G8270, SIGMA, St. Louis, MO, USA). Finally, the degree of hydrolysis is expressed as the percentage of total sugars in the first supernatant at a given time to the initial weight of dry starch, also denoted as dextrose equivalent (DE).

### 2.5. Starch Purity

The purity of the extracted starch was detected using the K-TSTA-100A 02/22 kit (Megazyme International Ireland Ltd., Co Wicklow, Ireland), a process outlined in both AOAC 996.11 and AACC method 76-13.01.

### 2.6. Amylose and Amylopectin Ratio

The procedure was carried out under the guidelines in the protocol of the K-AMYL 06/18 kit from Megazyme^®^ (Megazyme International Ireland Ltd., Co Wicklow, Ireland). The basis of this kit is the modified method of amylose detection by precipitation of amylopectin with Concanavalin A previously developed by Yun & Matheson (1990) [38].

### 2.7. Optical Microscopy

Native and modified jicama starch (0.1 g) was suspended in 1 mL of Tween 80 (10099956, SIGMA, St. Louis, MO, USA), then 1 mL glycerol (G9012, SIGMA, St. Louis, MO, USA) was added, vortexed for 5 s, and processed for 60 s in a Branson-1510 ultrasonic bath (Danbury, CT, USA). Images were then captured using a Nikon Eclipse 50i microscope (NIKON Instruments INC., NY, USA), equipped with a DS-U3 digital camera (NIKON Instruments INC., NY, USA), with the aid of NIS-Elements F 2.30 software (NIKON Instruments INC., NY, USA). In contrast, the observation of electrosprayed starch microspheres was conducted directly on the slide. Finally, based on these images, approximately 100 particles were analyzed for some shape descriptors such as perimeter, area, roundness, and Feret (Fe) diameters with ImageJ software (https://imagej.net/ij/download.html, accessed on 14 January 2025, NIH, MD, USA).

### 2.8. Scanning Electron Microscopy

Native and modified starches were structurally characterized using micrographs obtained with an environmental scanning electron microscope (FEI Quanta, FEG 250, Hillsboro, OR, USA) at 5 kV with the back-scattered electron detector. Sample preparation was performed by directly sprinkling dried native and modified starch onto aluminium trays provided with a double-sided carbon tape.

### 2.9. Differential Scanning Calorimetry

The thermal properties of starch were evaluated by weighing 2.5 mg of jicama starch in an aluminum capsule, adding 7 μL of distilled water, and then hermetically sealing the capsule [39]. Samples were equilibrated for 24 h at room temperature for subsequent analysis in a differential scanning calorimeter (DSC Q2000, TA Instruments, New Castle, DE, USA), heating at a rate of 5 °C/min [40] over a temperature range of 20 to 120 °C [25]. The glass transition (Tg), characterized by the onset (To), peak (Tp), termination (Te), and enthalpy change (ΔH) temperatures, was obtained from the heat absorption endotherm using TA Universal Analysis 2000 software (TA Instruments, New Castle, DE, USA).

### 2.10. Fourier Transform Coupled Infrared Spectroscopy

Spectra were obtained by placing the powdered starch (~0.1 g, 25 °C) directly on the surface of a Cary 630 portable FTIR spectrometer (Agilent Technologies Inc., Santa Clara, CA, USA) equipped with a single-bounce attenuated total reflectance (ATR) diamond crystal interface. Spectra were collected in the 4000–650 cm^−1^ spectral region with a resolution of 4 cm^−1^ by pressing the powder sample onto the crystal using a pressure clamp. The absorbance spectrum was obtained by comparing the spectrum of the sample with that of a blank optical path (reference spectrum). The analysis of spectral data was conducted using Spectragryph software (v1.2.16.1, Spectragryph, Germany).

### 2.11. X-Ray Diffraction

The starch samples were placed in the sample holder, and diffraction patterns were obtained (40 kV, 15 mA, CuK, λ = 0.154 nm, 5–60° (2θ°)). To evaluate the degree of crystallinity, Equation (1) is used according to González-Lemus et al. (2018) [25], where *C_R_*, *A_C_*, and *A_a_* represent the relative crystallinity, the crystalline area, and the amorphous area, respectively.

(1) $$C_{R}=\frac{A_{c}}{A_{c}+A_{a}}*100$$

### 2.12. Visco-Amylographic Profile

**Figure 1:**
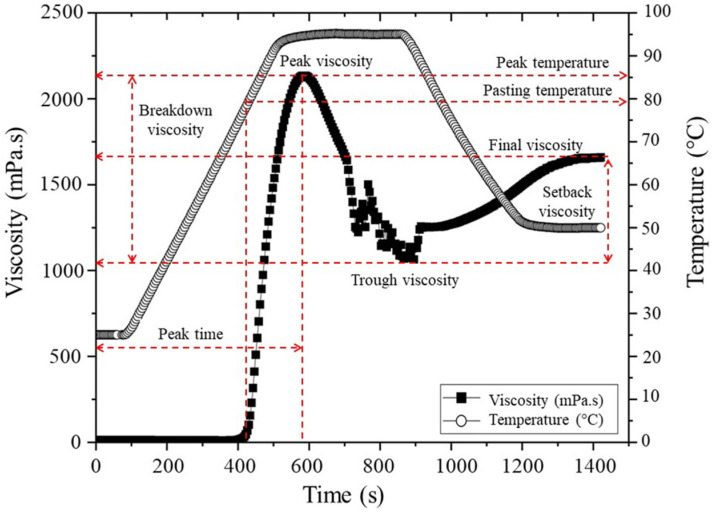
HR-3 rheometer starch viscoamylographic profile. Own data curve. Tags adapted from [41], polymers, 2025.

Viscosity profiles were obtained based on Contreras-Jiménez et al. [12] with some modifications. In brief, 2 g of native and modified starch powder was weighed and suspended in 18 mL of distilled water before being placed in the starch cup of the rheometer to perform the test. The curves were obtained in a Discovery HR-3 rheometer (TA Instruments, New Castle, DE, USA) using a Starch cell geometry (Starch cell Aluminium-999188), with a constant rotation at 16 rad.s^−1^. Initially, the temperature was stabilized at 25 °C for 60 s, after, the temperature was increased at a rate of 10 °C/min to 95 °C, held for 360s, finally cooled at a rate of 10 °C/min to 50 °C, where it was held for 300 s. Data were analyzed in the TA Instruments Trios (4.0.2.30774) software to obtain the pasting parameters shown in Figure 1. As illustrated in the diagram, the pasting temperature (PT) denotes the minimum temperature necessary for the baking of a sample obtained at the onset of the viscosity increase. The peak viscosity (PV) corresponds to the maximum viscosity observed at the equilibrium point between swelling and leaching of the polymer. Furthermore, the peak temperature (PkT) is the temperature at PV. Collectively, these parameters signify the water-binding capacity of the starch. Trough viscosity (TV) is defined as the minimum viscosity obtained after the peak viscosity during the cooking process. The difference between PV and TV is known as breakdown viscosity (BDV), which is a measure of the degree of disintegration of the starch granules when subjected to temperature and shear force. The final viscosity (FV) measures the viscosity of the gel after cooking and cooling, and indicates its stability at the end of this process. Finally, the setback viscosity (SBV) is given by the equation SBV = FV-TV and refers to the retrogradation of the starch molecules during cooling [41,42].

### 2.13. Preparation of Electrosprayed Solutions

Based on the design of experiments for microsphere synthesis, starch dispersions were prepared at the required concentration (*w*/*v*), subsequently heated to boiling, and maintained at this temperature for 10 min to achieve a complete gelatinization under constant stirring in a heated Super-Nuova Multi-Place stirrer (Thermo Scientific) then was cooled to room temperature and poured into hypodermic syringes for further use.

### 2.14. Apparent Viscosity

Apparent viscosity for electrosprayed solutions was measured according to Li, Kong, and Ziegler (2020) [43], using a Discovery HR-3 hybrid rotational rheometer (TA Instruments, New Castle, DE, USA) using a parallel plate geometry with 40 mm diameter (998807 TA Instruments, Elstree, UK) and determined from flow curves with a shear rate of 0.1 at 100 s^−1^, at 20 °C. The flow curves were fitted to a model using TRIOS TA Instruments software (4.0.2.30774) and the “best flow fit (Stress vs. Rate)” analysis tool, choosing the model with the best fit and its parameters. Also, the apparent viscosity was introduced in the design of experiments to evaluate the impact of acid hydrolysis and starch concentration on the viscosity of the electrosprayed solutions using Design Expert V9 software (Stat-Ease In., Minneapolis, MN, USA).

### 2.15. Surface Tension

The surface tension (γ) of the electrosprayed solutions was determined using a K6-Krüss tensiometer (Hamburg, Germany), which is based on measuring the tensile strength of a stretched sheet until it breaks. The samples were analyzed in triplicate at room temperature following the method described by the manufacturer. The surface tension value is obtained directly from the equipment in mN/m units. The effect of starch hydrolysis on the surface tension of the electrosprayed solutions was evaluated using Design Expert V9 software (Stat-Ease In., Minneapolis, MN, USA) based on the design of experiments used for microsphere synthesis.

### 2.16. Electrical Conductivity

The quantification of this property was conducted using a ST20 Starter conductivity meter (OHAUS, Parsippany, NJ, USA) with an accuracy of 0 to 20 mS under the manufacturer’s instructions. Briefly, the electrode is rinsed in water, dried with a paper towel, and inserted into the measuring solution to obtain the electrical conductivity value.

### 2.17. Electrospray Synthesis of Jicama Starch Microspheres

The synthesis of jicama starch microspheres with varying degrees of hydrolysis was conducted using a basic electrospraying apparatus (CICATA-IPN, Queretaro, Mexico), which comprises a vertical syringe pump, a power source, and a single capillary nozzle, previously employed by Rentería-Ortega et al. (2020) [44]. To evaluate the effect of the degree of hydrolysis, a design of experiments using response surface methodology was developed, following a central composite design with 6 replicates at the central point, resulting in a total of 20 runs (Table 1). The design expert software (v.9) was employed. The parameters evaluated encompassed hydrolysis time (1 to 24 h), within a range designated as rapid hydrolysis [20], starch concentration (6.6 to 23.4% *w*/*v*), and applied voltage (8.30 to 16.70 kV). These parameters were adjusted following a comprehensive literature review on the starch utilization as a wall material in diverse electrospraying methodologies [27,28,29,30,31,32,33,45]. Furthermore, the flow rate was set at 2 mL/h for all experiments according to the equipment characteristics.

### 2.18. Statistical Analysis

Results are presented as arithmetic mean plus/minus sample standard deviation from at least three replicates in each experiment. All analyses were made in MYSTAT software (SYSTAT 13, http://systatsoftware.com/downloads/download-mystat/ consulted on 19 October 2021, Inpixon, Palo Alto, CA, USA). On the other hand, the electrospray starch microspheres synthesis data were evaluated using Design Expert 9 software (Stat-Ease Inc., MN, USA).

## 3. Results and Discussion

### 3.1. Physicochemical Characterization of Jicama (P. erosus) Native Starch

As illustrated in Table 2, the physicochemical characterization of native jicama starch shows that the extraction process is suitable for this material, as a high-purity material was obtained, along with a low concentration of maltodextrins and glucose. These values of glucose and maltodextrins could have been produced by starch damage during extraction [46]. Conversely, the amylose content is consistent with the range reported for certain jicama varieties [23] and analogous to values obtained in other studies (23.6%, 25.2%, and 26.4%, respectively) [12,24,47] and is considered, due to its amylose content, a normal starch [46]. The percentage of amylose is important because it has been demonstrated to impact the structure of starch, the viscosity of the resultant gel, and the susceptibility or resistance to acid hydrolysis [48,49].

### 3.2. Morphology and Feret Diameter of Native Jicama Starch

**Figure 2:**
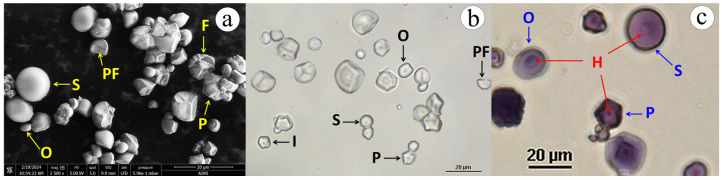
ESEM (**a**) and light microscopy (**b**,**c**) micrographs of jicama (*P. erosus*) starch granules. S = spherical, O = oval, P = polyhedral, I = irregular, PF = pressure facet, F = fragmented or damaged granule, H = hilum.

The jicama starch granule (Figure 2) showed spherical, polygonal, and oval compacted shapes, which are similar to the findings reported by Martínez-Bustos et al., (2006) [50]. Regardless of its shape, the native jicama starch granules (Figure 2c) had a closed and dotted hilum; the surface texture was smooth, but exhibited facets resulting from pressure; some broken granules were also observed [51]. In comparison, the Feret diameter of native jicama starch ranged from 3.5 to 19.5 µm, which is consistent with the findings reported in other studies [24,25]. This indicates that the jicama starch has a narrower size distribution compared to potato starch (5 to 100 µm) and slightly smaller granules than those observed in maize (3 to 30 µm) [46].

### 3.3. Hydrolysis of Jicama Starch

The hydrolysis progression of jicama starch during the first 24 h (Table 3) reveals a maximum of 42.7% hydrolysis degree, following a potential behavior (R^2^ = 0.9825), a result analogous to that of mung bean starch under the same hydrolysis conditions, agreeing that both jicama and mung starches possess the same C-type polymorphism [23,49]. It is suggested in the literature that a phase known as fast hydrolysis occurs during this period, involving the breakdown of the outer amorphous region (amylose chains with a degree of polymerization > 300) and the intra-cluster crystalline zone (long amylopectin chains) [14,20,49]. On the other hand, starch hydrolysis products with a dextrose equivalent (DE) less than 20 are classified as maltodextrins; in this study, they were produced within the first 12 h, after which starch hydrolysis products were designated as syrup solids [21].

### 3.4. Effect of Hydrolysis on Morphology and Jicama Starch Feret Diameter

**Figure 3:**
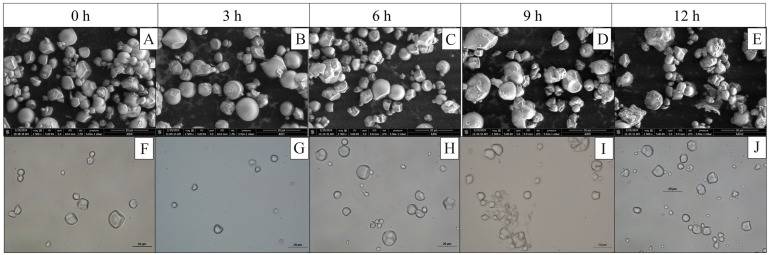
Effect of H_2_SO_4_ hydrolysis time on morphometric jicama starch characteristics. (**A**–**E**): SEM micrographs; (**F**–**J**): brightfield micrographs.

**Figure 4:**
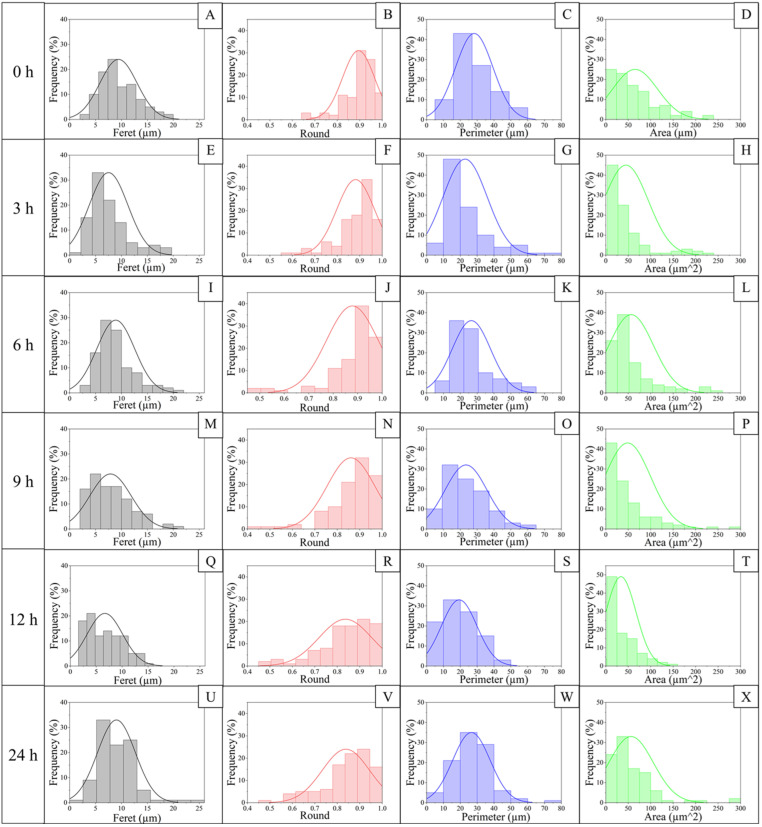
Morphometric parameters according to the hydrolysis degree of jicama starch. Feret distributions are comprised on (**A**,**E**,**I**,**M**,**Q**,**U**) from jicama starch native (0 h) and hydrolyzed for 3, 6, 9, 12, and 24 h respectively. Round distributions in (**B**,**F**,**J**,**N**,**R**,**V**) from jicama starch native (0 h) and hydrolyzed for 3, 6, 9, 12, and 24 h respectively. Perimeter distributions are presented in (**C**,**G**,**K**,**O**,**S**,**W**) from jicama starch native (0 h) and hydrolyzed for 3, 6, 9, 12, and 24 h respectively. Area distributions are comprised in (**D**,**H**,**L**,**P**,**T**,**X**) from jicama starch native (0 h) and hydrolyzed for 3, 6, 9, 12, and 24 h respectively. Frequency distributions were expressed as a percentage of about 100 particles analyzed.

The micrographs illustrating the changes in the starch granules’ morphology after hydrolysis (Figure 3) showed that between 3 and 6 h of hydrolysis, superficial erosion is observed, subsequently followed by the formation of cracks in what could be the amorphous zone of the granules. At 12 h, there is an increase in the presence of granules with cracks, accompanied by the release of granule fragments (2 < Fe < 4 µm) (Figure 4). In addition, spherical and oval granules demonstrate greater integrity than polygonal and irregularly shaped granules.

Regarding the morphometric parameters (Figure 4), the roundness of the granules decreases during this process, which may be associated with their fragmentation, generating an irregular morphology that also modifies the perimeter. On the other hand, both perimeter and area show a distribution that widens at 3 h and narrows towards 12 h of hydrolysis. This may be associated with swelling and subsequent fragmentation and fracture of the granule. This is followed by a release of fragments between 12 and 24 h of hydrolysis, which may then agglomerate. These changes may be associated with how hydrolysis occurs in the starch structure. The literature indicates that the process starts in the amorphous region of the granule, followed by erosion in the crystalline area [46,52]. There are two mechanisms described for this process; for type A starches, such as corn starch, the channels running from the surface inwards can provide an initiation zone for attack, being different for type B starches such as potato starch, where the process of erosion occurs, resulting in the generation of channels that widen as hydrolysis progresses [20,53]; in this case, the jicama starch displaying characteristics of both types A and B starches must follow both breaking processes, but with some granules remaining unfragmented.

### 3.5. Thermal Properties of Jicama Starch and Its Hydrolysates

The changes in the thermal parameters of jicama starch as the hydrolysis time elapses are shown in Table 4. It is well known that when starch is heated at high temperature with a water content above 60% (*w*/*v*), the gelatinization process, detectable from DSC endotherms [54], occurs. In this process, the semi-crystalline structure of starch is transformed into an amorphous one, due to the disruption of the molecular order (rupture of hydrogen bonds) reflecting in the gradual loss of the granular structure in an irreversible way [24,46], as well as the change from a slurry into a semi-solid or gel with significant viscous properties [55]. In the case of native jicama starch, we see that the values of peak temperature (Tp) are very similar to those reported by Ramírez-Miranda et al., (2017) [24] and by González-Lemus et al., (2018) [25], where variations in these values regularly depend on cultivar, extraction method, place of origin, amylose content, degree of amylopectin cross-linking, starch crystallinity, chain length distribution, concentration of components (fats, proteins, etc.) and amount of damaged starch [23]. As the hydrolysis process proceeds, the Tp shows the highest value at 6 h, possibly suggesting the formation of stronger intermolecular bonds or increased molecular order or crystallinity [56]. Subsequently, a decrease is perceived at 12 h and no endotherm is formed at 24 h. On the other hand, the enthalpy change (ΔH) reflects the amount of double helix order [17]. For native starch, its value is around that reported again by González-Lemus et al., (2018) [25] and Shi, et al. (2021) [57]. During the hydrolysis process, ΔH remains unchanged within the first 6 h, decreases significantly at 12 h, and ceases to be perceived at 24 h, given the absence of an endotherm (Table 5). This behavior is characteristic of the acid hydrolysis process of granular starch [56]. This may indicate the gradual loss of order in the crystal structure and the variation in the bond strengths as there is a simultaneous attack on amylose and amylopectin in the initial phase of hydrolysis [23], while the absence of the endotherm could be indicating less amount of double helix and a less-ordered crystal structure [17,48].

### 3.6. Fourier Transform Infrared Spectroscopy

**Figure 5:**
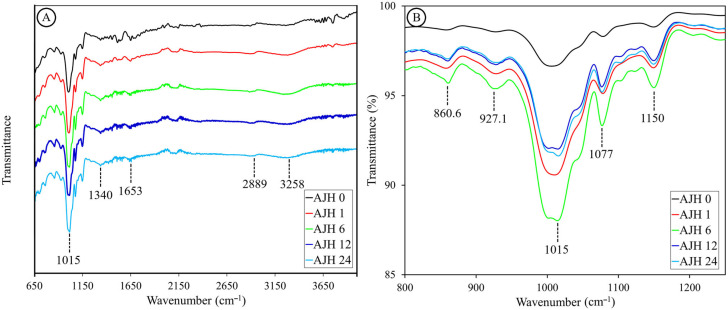
FTIR spectra of native jicama starch (AJH0) and subjected to acid hydrolysis for 1 (AJH1), 6 (AJH6), 12 (AJH12), and 24 h (AJH24). (**A**) shows the spectra from 650 to 4000 cm^−1^, whereas (**B**) shows the spectra in the carbohydrate region (800 to 1250 cm^−1^).

The spectra of both native starch and the hydrolysates analyzed (Figure 5A) show a similar behavior to that reported by Wigati et al., (2022) [58] for native jicama starch. All spectra display a peak at 3258.6 ± 17.1 cm^−1^, ranging from 3000 to 3500 cm^−1^, associated with the hydrogen bonds of water (O-H stretching). In both native and hydrolyzed starch, a prominent peak is present around 2889 cm^−1^ in the region corresponding to the C-H bond vibration, where the change in intensity has also been related to the progress of starch retrogradation [59]. Subsequently, a consistent, but small, peak is found in all samples around 1653 cm^−1^, which may be associated with conjugated carbonyl and carboxyl groups and C-O vibrations, or an interaction between water and starch. On the other hand, a peak is detected around 1340 cm^−1^, indicating O-H stretching. Its intensity decreases with hydrolysis, reaching its lowest point at 6 h and rising slightly to remain at 12 and 24 h. Regarding the characteristic region of carbohydrates (1200 to 800 cm^−1^), shown in Figure 5B, both in native and hydrolyzed starch, a peak at 1149 cm^−1^ can be distinguished, which could be associated with a C-O stretching of sucrose confirmed by the presence of a peak around 926 cm^−1^, this molecule may come from the starch extraction process where there was no complete purification, and would help explain the peak around 860 cm^−1^, which may be associated with a C-C stretching of fructose produced during the hydrolysis of sucrose or some fructooligosaccharide. However, this contrasts with the fact that these substances are soluble in water and could have been removed by washing during hydrolysis.

Additionally, there is a peak at 1078 cm^−1^ related to C-O and C-C stretching and C-O-H bending, while the most prominent peak is found at 1003 cm^−1^ in native starch; in hydrolyzed jicama starch, it shifts towards 1014 cm^−1^. This peak is associated with the amorphous zone [60], where its intensity decreases in the hydrolyzed sample during 6 h and increases at 12 and 24 h. On the other hand, the FTIR technique allows for the visualization of the double helix degree through the intensity ratio at 995/1022 cm^−1^ and the degree of order through the intensity ratio at 1047/1022 cm^−1^. Regarding this, in Table 5 “IR ratios (FTIR parameters)”, it is observed that the degree of double helix is maintained up to 12 h increases slightly at 24 h. Finally, the degree of order increases in hydrolyzed jicama starch with a peak at 6 h and decreases to remain stable at 12 and 24 h of hydrolysis. Compared with the ΔH data, we see that the degree of order measured by the double helix dissociation [17] tends to decrease. This could be related to the sensitivity of the method, where DSC may not measure the degree of order of the short chains generated during acid hydrolysis [52].

### 3.7. X-Ray Diffraction of Native and Hydrolyzed Jicama Starch

**Figure 6:**
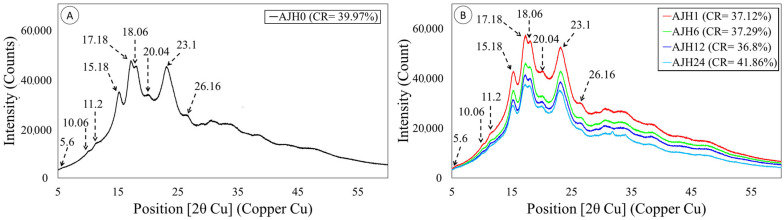
Diffraction patterns of jicama starch. (**A**) native starch (AJH 0) (left); (**B**) acid hydrolyzed modified starch 1h (AJH 1), 6h (AJH 6), 12h (AJH 12), and 24 h (AJH 24), and their respective behavior at peaks 5.6° (1), 10.6° (2), 11.2° (3), 15.18° (4), 23.1° (7), 26.16° (8), and the duplex at peaks 17.18° and 18.06° (5). The relative crystallinity percentage is shown with the symbol CR.

X-ray diffraction results (Figure 6A) of native jicama starch show weak peaks at 5.6°, 10.06°, 11.2°, 20.4°, and 26.16° (2θ), strong peaks at 15.18° and 23.1° (2θ), and a duplex at 17.18° and 18. 06° (2θ). The results mentioned above are characteristic values for a legume starch C-type diffraction pattern [46], coinciding with that reported by several authors [12,24,25]. On the other hand, it is observed that the relative crystallinity in the modified jicama starch shows a decrease until a minimum detected at 12 h of acid hydrolysis, while at 24 h it increases again, while the peaks are conserved in all treatments (Figure 6B). The factors affecting the degree of crystallinity include the amylopectin content and the orientation and size of the double helices in the crystalline area [23]. In turn, how the acid attacks the starch structure may be associated with the diffraction pattern [53]. In this sense, starches with a C-type diffraction pattern tend to have an intermediate susceptibility to hydrolysis compared to those of type A (more resistant) and type B (less resistant) [20,49].

### 3.8. Viscoamylographic Profile Changes in Jicama Starch by Acid Hydrolysis

The native jicama starch presents a typical behavior of starches, forming a curve that starts with the minimum temperature required for granule swelling, reaching a pasting temperature (PT) of 76 ± 1 °C. Following, the maximum viscosity observed at the equilibrium point between swelling and leaching of the polymer is a peak viscosity (PV) of 2137.4 ± 98.4 mPa.s, which corresponds to the peak temperature (PkT) of 94.6 ± 0.1 °C. Collectively, these parameters signify the water-binding capacity of the starch [41,42]. Then, the minimum viscosity obtained after the peak viscosity during the cooking process, the trough viscosity (TV), was 1003.1 ± 43.2 mPa.s. It is associated with the total rupture of the granule during gelatinization, and attainment a breakdown viscosity (BDV) of 1134.3 ± 58.9 mPa.s. The native jicama starch also showed a setback viscosity (SBV), relative to retrogradation process, of 658.4 ± 61.4 mPa.s and a final viscosity (FV) of 1661.5 ± 102.8 mPa.s (Table 5). The values obtained are very similar to those reported by Contreras-Jiménez et al. (2019) [12], and it is important to note that viscosity is concentration-dependent for starches and dextrins [61,62].

This curve provides insight into the behavior of jicama starch during the gelatinization process where, due to the action of moisture and temperature, the granule structure collapses, the crystallites melt, the double helixes unwind and hydrogen bonds break [58], and then by association and formation of bonds between chains, a continuous network or gel is created [61]. This process is shown to be different for hydrolyzed jicama starch in that pasting temperature (PT) remains unchanged up to 12 h (74.8 ± 4.5 °C) and decreases significantly (*p* ≤ 0.05) at 24 h (62.8 ± 4.2 °C). In turn, PV, TV, BDV, SBV, and FV decrease by 97.7 ± 0.4% during the first hour of acid treatment related to native starch (0 h), and remain without significant differences afterwards, regardless of the degree of hydrolysis achieved in the time studied. On the other hand, PkT, which together with PV indicates the water holding capacity of starch before granule rupture during swelling, is around 82.7 ± 1.7 °C for jicama starch hydrolyzed between 0 and 12 h of hydrolysis and decreases to 69.5 ± 1.2 °C at 24 h. These results are in agreement with those reported by Chen et al. (2023) [62], finding that acid hydrolysis strongly impacts starch swelling capacity and viscosity development, which can be associated with the shortening of amylose and amylopectin chains and modification of crystallinity during hydrolysis [62].

### 3.9. Effect of Jicama Starch Hydrolysis Time and Starch Concentration on EHDA Solution Physical Properties

#### 3.9.1. Apparent Viscosity (η), and Rate Index (n)

**Figure 7:**
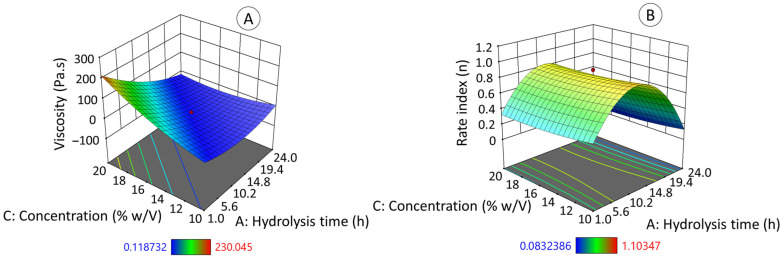
Effect of acid hydrolysis degree of jicama starch on apparent viscosity (**A**) and rate index (**B**) as a function of hydrolysis time and starch concentration.

Figure 7 presents the surface response graphics of apparent viscosity (Figure 7A) and rate index (Figure 7B). These values (η, n) were obtained from the flow curves of the native and hydrolyzed jicama starch solutions, which were fitted to the Herschel–Bulkley model, in which the sample presents an initial yield stress, followed by a shear thinning behavior. The specific data results of this test are shown in Table S1 of the Supplementary Material.

The graphs (Figure 7A,B) show that both the hydrolysis time (directly related to the hydrolysis degree) and the concentration (*w*/*v*) at which the EHDA solutions were prepared had a direct impact on the value of the apparent viscosity (Figure 7A) while only the hydrolysis time determined the behavior of the rate index (Figure 7B). According to Kanyuck et al. (2019) [61], the formation of the maltodextrin gel network during starch acid hydrolysis occurs in two stages: initially through the helical association of two chains and subsequently, with aggregation between the double helices into crystallized regions and longer chains connecting the aggregates of helices into a gel. In the present work, acid hydrolysis affected the swelling capacity and viscosity development of starch. Concentration also plays an important role in the rheology of jicama starch gels (based on native and hydrolyzed), as expected [63].

The apparent viscosity (η) and the rated index (n) equations follow a quadratic model (Equation (2), R^2^_ADJ_ = 0.8951 and Equation (3), R^2^_ADJ_ = 0.7387); both mathematical models are statistically significant (*p* ≤ 0.05).

η = 0.28A^2^ + 0.89C^2^ − 57.23AC + 2.96A − 5.06C + 41.41 (2)

n = −0.63A^2^ − 0.14A + 0.83 (3)

where A refers to hydrolysis time (hours), B to voltage (kV), and C (% *w*/*v*) to starch concentration.

#### 3.9.2. Surface Tension (γ)

**Figure 8:**
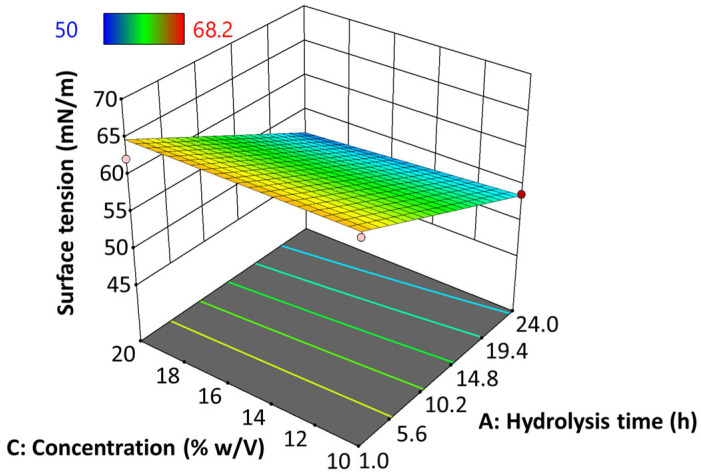
Effect of hydrolysis time of jicama starch on the surface tension of an EHDA solution.

Figure 8 presents the behavior of the surface tension (γ) of native and hydrolyzed starch EHDA solutions, when varying hydrolysis time and hydrolyzed starch concentration. In this case, data was adjusted to a linear model (Equation (4), *p* ≤ 0.05) with an adjusted determination coefficient (R^2^_ADJ_) of 0.7714.

γ = −6.10A − 0.4750AC − 0.5072C + 58.81(4)

where A refers to hydrolysis time (hours), B to voltage (kV), and C (% *w*/*v*) to starch concentration.

The results show that the impact of hydrolysis time on surface tension is predominant over concentration, such that γ tends to decrease as the degree of starch hydrolysis increases. The specific data results of this test are shown in Table S1 of the supplementary material.

#### 3.9.3. Electrical Conductivity

**Figure 9:**
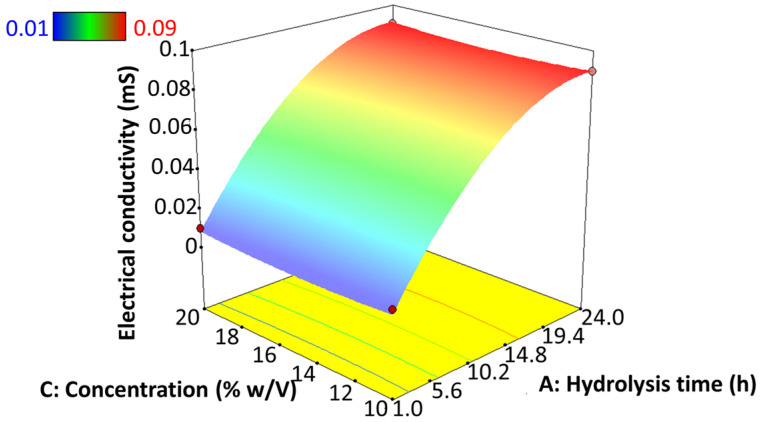
Effect of hydrolysis time of jicama starch on the electrical conductivity of an EHDA solution.

Electrical conductivity is a pivotal parameter in electrohydrodynamic atomization, since the driving force is the charge exerted on the solutions for polymer elongation as well as for jet breakdown. The presence of charge carriers, such as ions or polarized molecules, becomes a relevant factor in dielectric materials, for example, starch [64]. This property is often influenced by the concentration and type of polymer, solvent, and the availability of ionizable compounds [26]. However, Figure 9 shows that the electrical conductivity is directly influenced by the degree of hydrolysis of the starch, regardless of the starch concentration used in the solution. In this sense, the conductivity tends to increase as the hydrolysis progresses. Its highest point was found at 24 h of hydrolysis. On the other hand, the best-like microsphere particle formation was obtained at a low hydrolysis degree when low electrical conductivity is present. This contrasts with studies based on potato starch solubilized in formic acid, where an increase in exposure time led to a decrease in electrical conductivity [65]. These authors also reported that electrical conductivity does not affect the morphology and diameter of the fibers synthesized by EHDA [26]. The specific data results of this test are shown in Table S1 of the supplementary material.

### 3.10. Morphometric Characteristics of the Electrosprayed Jicama Starch Particles

The effect of hydrolysis time on the Feret diameter (Fe) of the particles generated by EHDA (Figure 8) fits a quadratic model (Equation (5), R^2^_ADJ_ = 0.8698, *p* ≤ 0.05):

Fe = 602.4A^2^ + 376.4C^2^ − 884.5AC − 969.7A + 713.7C − 53.3 (5)

where A refers to hydrolysis time (hours), B to voltage (kV), and C (% *w*/*v*) to starch concentration.

**Figure 10:**
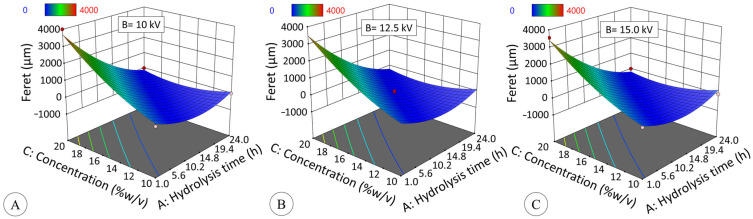
Effect of hydrolysis time (h) and starch concentration (% *w*/*v*) at 10 kV (**A**), 12.5 kV (**B**) and 15 kV (**C**) on the Feret diameter (μm) of modified starch particles generated by electrospraying. Data are fitted to quadratic model (*p* ≤ 0.05, R^2^ADJ = 0.8698) described by Equation (5).

Figure 10 shows that particle diameter is significantly influenced by concentration and hydrolysis time (*p* ≤ 0.05). Concentration and Feret diameter have a directly proportional relationship; however, this is true up to 6 h. In the modified starch for 12 (DE = 19.3) and 24 h (DE = 42.7), particle formation was not verified independently of solids concentration. It is known that the concentration of native starch influences the particle shape in electrospraying, in which high concentrations yield fibers, while low concentrations produce beads [9,66,67].

**Figure 11:**
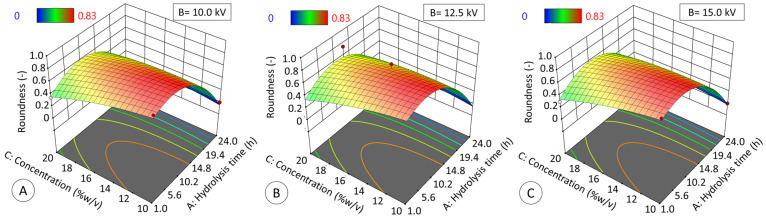
Effect of hydrolysis time (h) and starch concentration (% *w*/*v*) at 10 kV (**A**), 12.5 kV (**B**), and 15 kV (**C**) on the roundness of modified starch particles generated by electrospraying. The data obtained fit the quadratic model (R^2^ADJ = 0.9396, *p* ≤ 0.05) described by Equation (6).

Regarding roundness (RD, Figure 11), both hydrolysis time and solids concentration had a significant effect (*p* ≤ 0.05), adjusting data to a quadratic model (R^2^_ADJ_ = 0.9396, *p* ≤ 0.05):

RD = −0.39A^2^ − 0.05C^2^ + 0.12AC − 0.31A − 0.1C + 0.7 (6)

where A refers to hydrolysis time (hours), B to voltage (kV), and C (% *w*/*v*) to starch concentration. The specific data results of these both analyses, Feret and roundness, are shown in Table S1 of the Supplementary Material, and the bright field micrographs corresponding to the experimental design in Table 1 are shown in Figures S1 and S2.

On the other hand, although voltage did not have a significant effect on Feret diameter and roundness under the experimental conditions (*p* > 0.05), it is noteworthy that the application of voltage resulted in a modification of these parameters in comparison to the absence of voltage in the synthesis process. However, the effect of hydrolysis is more significant than the impact of voltage or solid concentration, as it modifies the structure of the starch and the properties of the gels. Consequently, the ability of jicama starch to be used as a coating material in the electrospray synthesis of particles or microspheres appears to be determined by the degree of hydrolysis.

## 4. Conclusions

Studies using starch as the only polymer are limited and require further investigation in electrohydrodynamic atomization and the food field [26]. In this regard, jicama could be a potential source of starch; however, this has not yet been tested in this technology [9]. Concerning their structural characteristics, starches encounter difficulties when employed as a sole wall material in EHDA applications. In an attempt to resolve this issue, studies have been conducted on the use of modified starches such as debranched starch, resistant maltodextrins, or solubilized in organic acids, among others. We hypothesize that the properties of jicama (*Pachyrhizus erosus*) starch can be modified by acid hydrolysis with sulfuric acid to improve its application in the EHDA process. We found that acid hydrolysis of jicama starch produced dextrins within 1 to 12 h with a degree of hydrolysis (expressed as dextrose equivalent (DE)) of less than 20%, and syrup solids at 24 h (DE of 40%) [45]. The FTIR results indicate only a polymer chain cut, and no functionalization was detected. The hydrolysis process mainly affected the water retention capacity of the starch, as well as the viscosity, surface tension, and electrical conductivity of the gels. In this regard, it is known that in EHDA, the polymer structure and solution properties impact the shape and size of the electrospray products [8]. In the present study, it was found that the surface tension and viscosity of jicama starch gels decrease as the degree of hydrolysis increases, although the electrical conductivity increases at longer times of acid hydrolysis, ranging from 0.01 to 0.09 mS. This may facilitate the separation of droplets during the electrospraying process, enabling the synthesis of particles from hydrolyzed jicama starch with a dextrose equivalent between 0.4 and 6.3. Additionally, the size and morphology were influenced more by the degree of hydrolysis than by the concentration of solids. However, with hydrolysis times of 12 and 24 h, it was not possible to form like-microspheres particles by electrospray under the conditions of the study. This indicates that jicama starch dextrins with a degree of hydrolysis below 6.3% can be used as the sole wall material to form particles by electrospraying.

## Figures and Tables

**Table 1:** Design of experiments to evaluate the effect of acid hydrolysis on the electrospray synthesis of jicama starch microspheres.

Starch Hydrolysis Time (h)	Starch Concentration (% *w*/*v*)	Voltage (kV)
1	10	10
1	10	15
1	20	10
1	20	15
6	15	12.5
12.5	6.6	12.5
12.5	15	8.3
12.5	15	12.5
12.5	15	12.5
12.5	15	12.5
12.5	15	12.5
12.5	15	12.5
12.5	15	12.5
12.5	15	16.7
12.5	23.4	12.5
24	10	10
24	10	15
24	20	10
24	20	15

**Table 2:** Chemical properties of native jicama starch.

Purity (g/100 g Starch)	Maltodextrins (g/100 g Starch)	D-Glucose (g/100 g Starch)	Amylose (g/100 g Starch)	Amylopectin (g/100 g Starch)	Humidity (% RH)
93.2 ± 2.9	7.5 ± 0.6	1.23 ± 0.04	20.2 ± 0.3	79.8 ± 0.3	10.1 ± 0.7

**Table 3:** Hydrolysis degree of jicama starch.

Hydrolysis Time (h)	Hydrolysis Degree (DE ^1^)
1	0.4 ± 0.1 ^a^
3	0.8 ± 0.1 ^a^
6	6.3 ± 0.3 ^b^
9	10.3 ± 0.2 ^c^
12	19.3 ± 0.5 ^d^
24	42.7 ± 0.7 ^e^

^1^ DE: dextrose equivalent (%). Different letters in the superscripts indicate significant differences (*p* ≤ 0.05).

**Table 4:** Effect of acid hydrolysis time of jicama starch on thermal properties and FTIR parameters.

Hydrolysis Time (h)	Tp	ΔH	IR Ratios (FTIR Parameters)		Relative Crystallinity
	(°C)	(J/g)	Double Helix Degree (995/1022)	Order Degree (1047/1022)	(%)
0	66.91 ± 0.12 ^a^	2.72 ± 0.06 ^a^	0.9985	1.0085	39.97
1	64.35 ± 0.06 ^b^	2.85 ± 0.18 ^a^	0.9985	1.0271	37.12
6	69.25 ± 0.22^c^	2.74 ± 0.44 ^a^	0.9988	1.0344	37.29
12	65.86 ± 0.17 ^d^	1.45 ± 0.21 ^b^	0.9986	1.0220	36.8
24	ND	ND	1.0019	1.0230	41.86

Tp: peak temperature; ΔH: gelatinization enthalpy; different letters in the superscripts indicate significant differences (*p* ≤ 0.05).

**Table 5:** Changes in the viscoamylographic profile of jicama starch as function of hydrolysis time.

Hydrolysis Time	PT	PV	PkT	TV	BDV	FV	SBV	Tp
(h)	(°C)	(mPa.s)	(°C)	(mPa.s)	(mPa.s)	(mPa.s)	(mPa.s)	(s)
0	76 ± 1 ^a^	2137.4 ± 98.4 ^a^	94.6 ± 0.1 ^a^	1003.1 ± 43.2 ^a^	1134.3 ± 58.9 ^a^	1661.5 ± 102.8 ^a^	658.4 ± 61.4 ^a^	410.2 ± 6.9 ^a^
1	75.4 ± 0 ^a^	58.5 ± 0 ^b^	84.8 ± 1 ^bc^	22.7 ± 0 ^b^	11.9 ± 0 ^b^	33.4 ± 0 ^b^	3.6 ± 0 ^b^	406.2 ± 0 ^a^
3	74.8 ± 0.6 ^a^	21 ± 1.9 ^b^	83.1 ± 1.2 ^bc^	15.2 ± 1.1 ^b^	5.8 ± 0.9 ^b^	20.9 ± 1.4 ^b^	5.7 ± 0.3 ^b^	398.1 ± 6.9 ^a^
6	76.8 ± 1.6 ^a^	16.1 ± 1.9 ^b^	83.8 ± 0.3 ^b^	11.5 ± 1 ^b^	4.5 ± 1 ^b^	16.5 ± 1.1 ^b^	4.9 ± 0.3 ^b^	412.5 ± 10.8 ^a^
9	73.7 ± 5.7 ^a^	13.3 ± 0.6 ^b^	80.8 ± 1.5 ^c^	9.9 ± 0.2 ^b^	3.4 ± 0.4 ^b^	14.4 ± 0.3 ^b^	4.5 ± 0.2 ^b^	397.3 ± 33.2 ^a^
12	74.8 ± 4.5 ^a^	13.8 ± 0.3 ^b^	81.2 ± 1.9 ^bc^	9.9 ± 0.1 ^b^	3.8 ± 0.3 ^b^	14.7 ± 0.4 ^b^	4.7 ± 0.3 ^b^	405.1 ± 29.6 ^a^
24	62.8 ± 4.2 ^b^	14.9 ± 0.2 ^b^	69.5 ± 1.2 ^d^	9.8 ± 0.1 ^b^	5.1 ± 0.3 ^b^	14.1 ± 0.1 ^b^	4.3 ± 0.2 ^b^	326.2 ± 24 ^b^

PT: pasting temperature; PV: peak viscosity at PkT; PkT: peak temperature; TV: trough viscosity; BDV: breakdown viscosity is equal to PV-TV; FV: final viscosity; SBV: setback viscosity is equal to FV-TV; Tp: peak time. Different letters in the superscripts indicate significant differences (*p* ≤ 0.05).

## Data Availability

The original contributions presented in the study are included in the article, further inquiries can be directed to the corresponding author.

## Supplementary Materials

The following supporting information can be downloaded at: https://www.mdpi.com/article/10.3390/polym17152069/s1, Table S1: Experimental design results from Table 1; Table S2: Bright field micrographs corresponding to the experimental design in Table 1; Figure S1: Jicama starch hydrolyzed-based solutions (24 h, 20% *p*/*v*) without voltage (a, b) and after electrospraying (c, d).
