# Supplementary material for: Synthesis of Jicama (Pachyrhizus erosus) Starch Particles by Electrospraying: Effect of the Hydrolysis Degree
Source: Polymers (Basel). 2025 Jul 29;17(15):2069. doi: 10.3390/polym17152069 (PMC12349585; doi:10.3390/polym17152069)
Supplement: Supplementary file 1 [file polymers-17-02069-s001.zip › polymers-3727265-supplementary.pdf]

Supplementary material from the article “Synthesis of jicama (Pachyrhizus erosus) starch particles by electrospraying. Effect of the hydrolysis degree.”

Table S1. Experimental design results from Table 1.

STD	RUN	Factors			Responses						
		A: Hydrolysis time	B: Voltage	C: Concentration	Viscosity	Yield stress	Rate Index	Surface tension	Feret	Roundness	Electrical conductivity
15	1	12.5	12.5	15	2.79	0.56	0.90	59.5	81.7	0.78	0.07
8	2	24	15	20	0.12	0.04	0.15	50.0	0.0	0.00	0.09
5	3	1	10	20	230.05	17.81	0.45	62.3	4000.0	0.35	0.01
14	4	12.5	12.5	15	2.79	0.56	0.90	59.5	121.3	0.68	0.07
16	5	12.5	12.5	15	2.79	0.56	0.90	59.5	123.9	0.65	0.07
1	6	1	10	10	1.14	0.28	0.39	64.2	238.2	0.83	0.01
11	7	12.5	16.7	15	2.79	0.56	0.90	59.5	81.0	0.77	0.07
18	8	12.5	12.5	15	2.79	0.56	0.90	59.5	121.7	0.68	0.07
9	9	6	12.5	15	34.54	6.67	0.52	68.2	215.9	0.72	0.02
10	10	12.5	8.3	15	2.79	0.56	0.90	59.5	91.0	0.72	0.07
7	11	1	15	20	230.05	17.81	0.45	62.3	3550.0	0.30	0.01
19	12	12.5	12.5	15	2.79	0.56	0.90	59.5	120.7	0.70	0.07
2	13	24	10	10	0.15	0.05	0.08	53.8	0.0	0.00	0.09
17	14	12.5	12.5	15	2.79	0.56	0.90	59.5	127.6	0.71	0.07
4	15	24	15	10	0.15	0.05	0.08	53.8	0.0	0.00	0.09
6	16	24	10	20	0.12	0.04	0.15	50.0	0.0	0.00	0.09
12	17	12.5	12.5	6.6	0.74	0.14	1.10	56.3	80.1	0.67	0.07
13	18	12.5	12.5	20	7.61	1.70	0.43	60.0	124.0	0.74	0.07
3	19	1	15	10	1.14	0.28	0.39	64.2	235.8	0.80	0.01

Table S2. Bright field micrographs corresponding to the experimental design in Table 1. This was employed to evaluate the effect of acid hydrolysis on the electrospray synthesis of jicama starch microspheres.

Starch hydrolysis Time (h)	Starch concentration (% w/v)	Voltage (kV)	Image 1	Image 2	Image 3	Image 4	Image 5
1	10	10	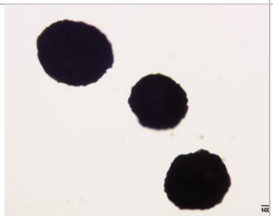	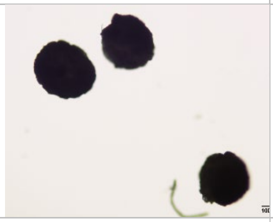	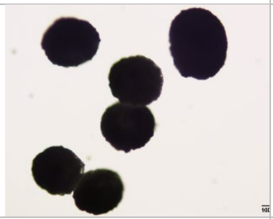	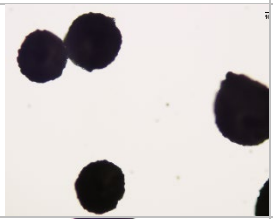	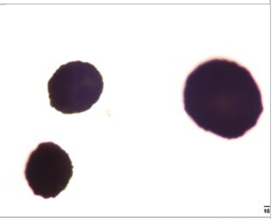
1	10	15	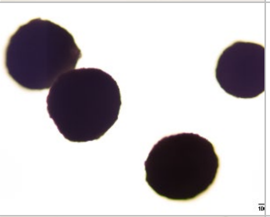	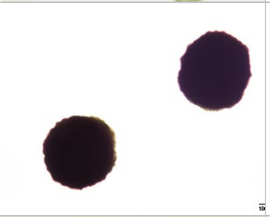	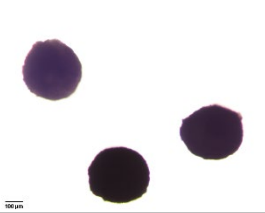	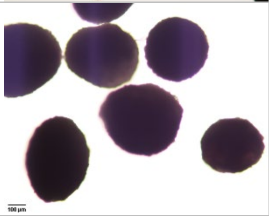	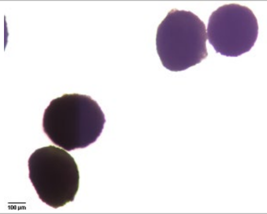
1	20	10	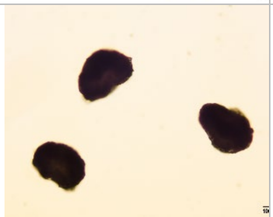	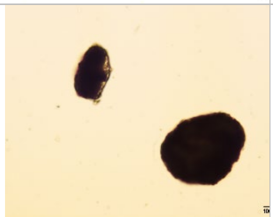	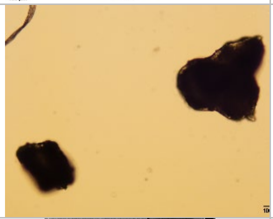	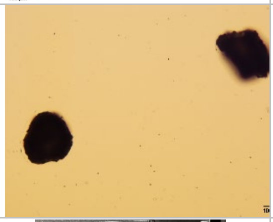	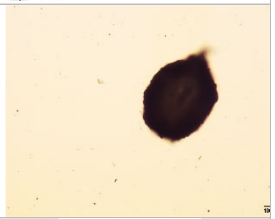
1	20	15	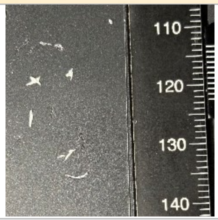	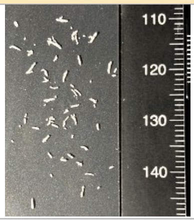	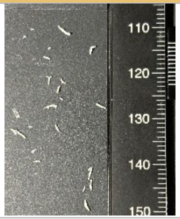	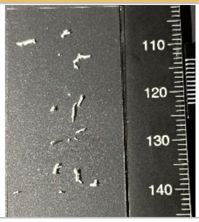	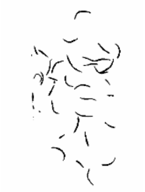

6	15	12.5	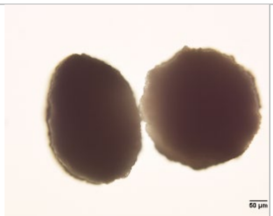	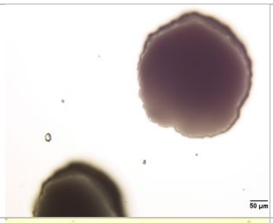	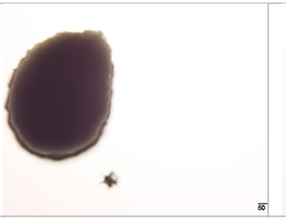	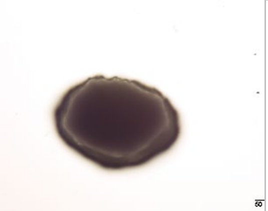	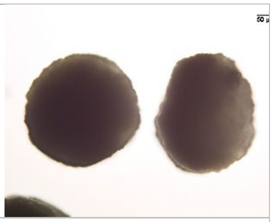
12.5	6.6	12.5	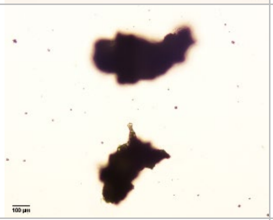	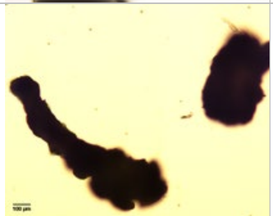	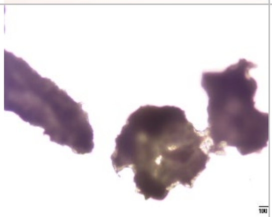	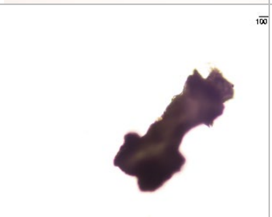	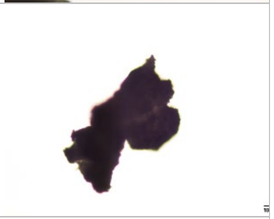
12.5	15	8.3	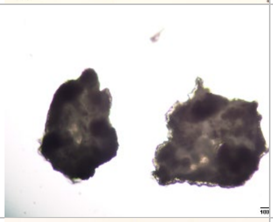	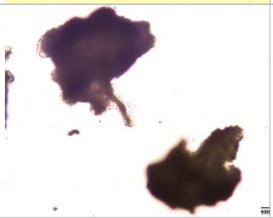	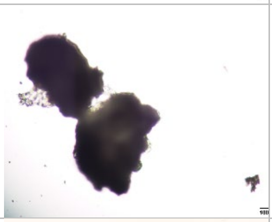	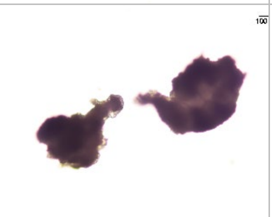	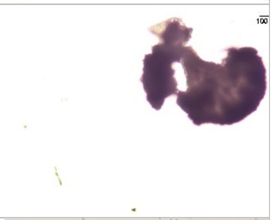
12.5	15	12.5	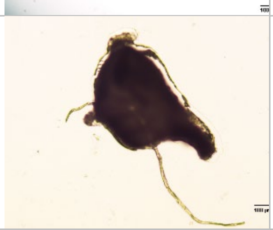	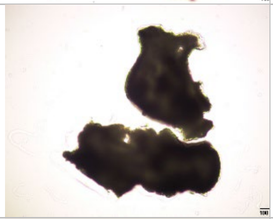	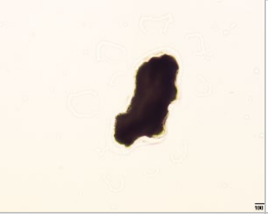	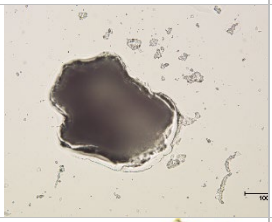	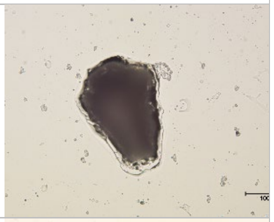
12.5	15	12.5	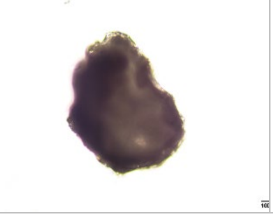	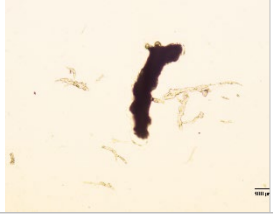	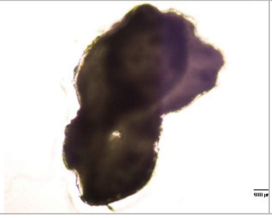	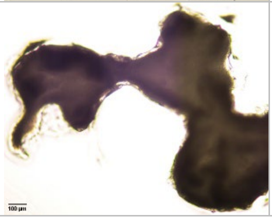	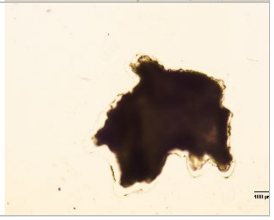

12.5	15	12.5	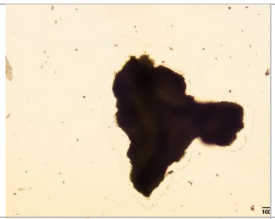	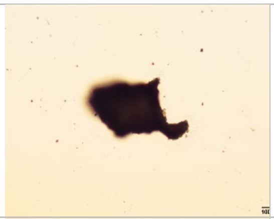	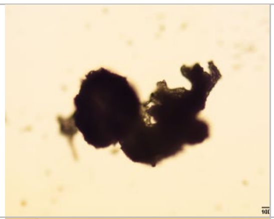	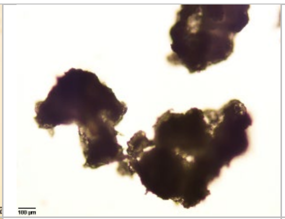	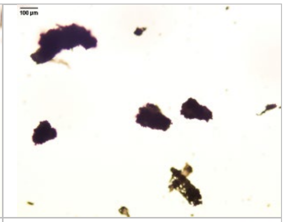
12.5	15	12.5	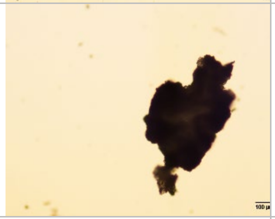	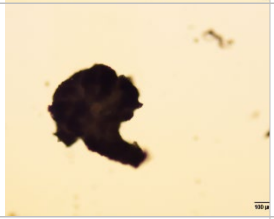	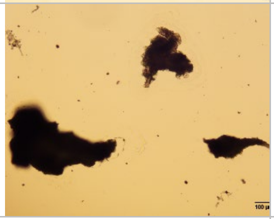	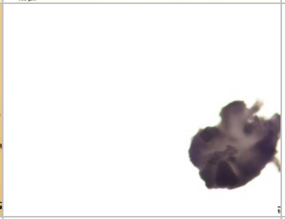	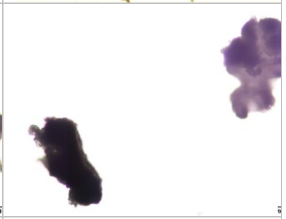
12.5	15	12.5	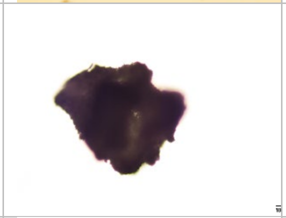	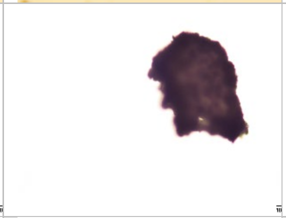	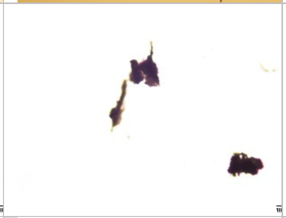	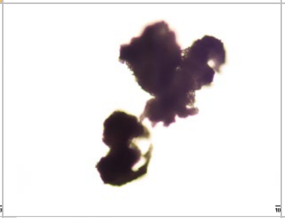	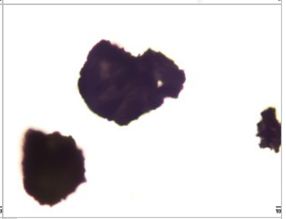
12.5	15	12.5	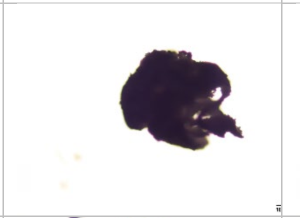	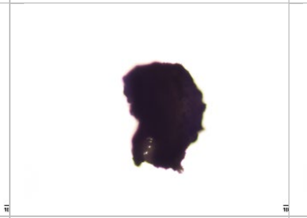	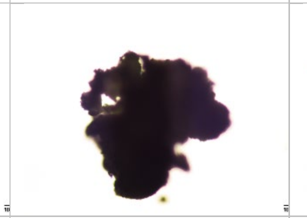	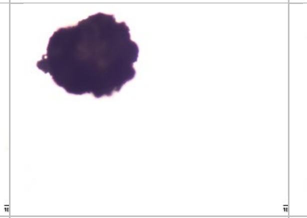	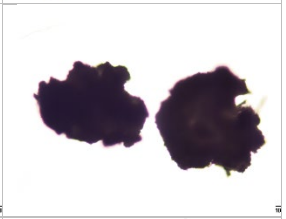
12.5	15	16.7	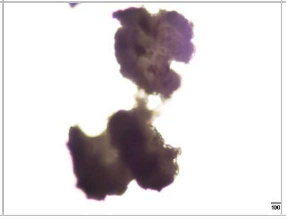	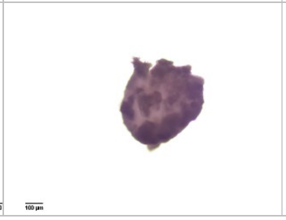	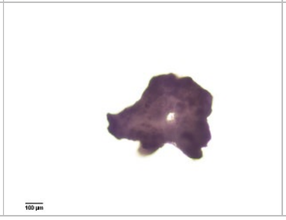	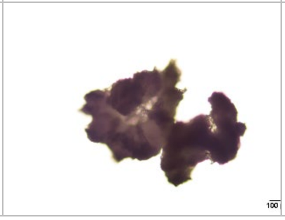	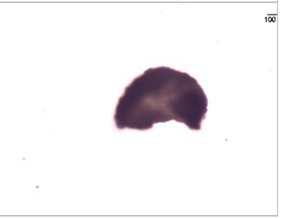

12.5	23.4	12.5	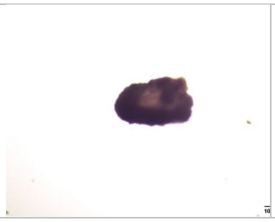	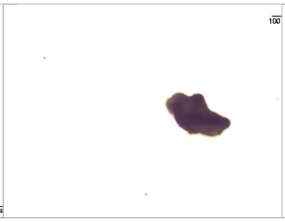	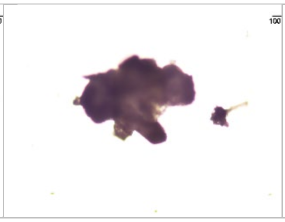	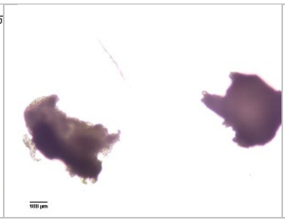	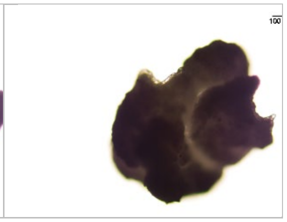

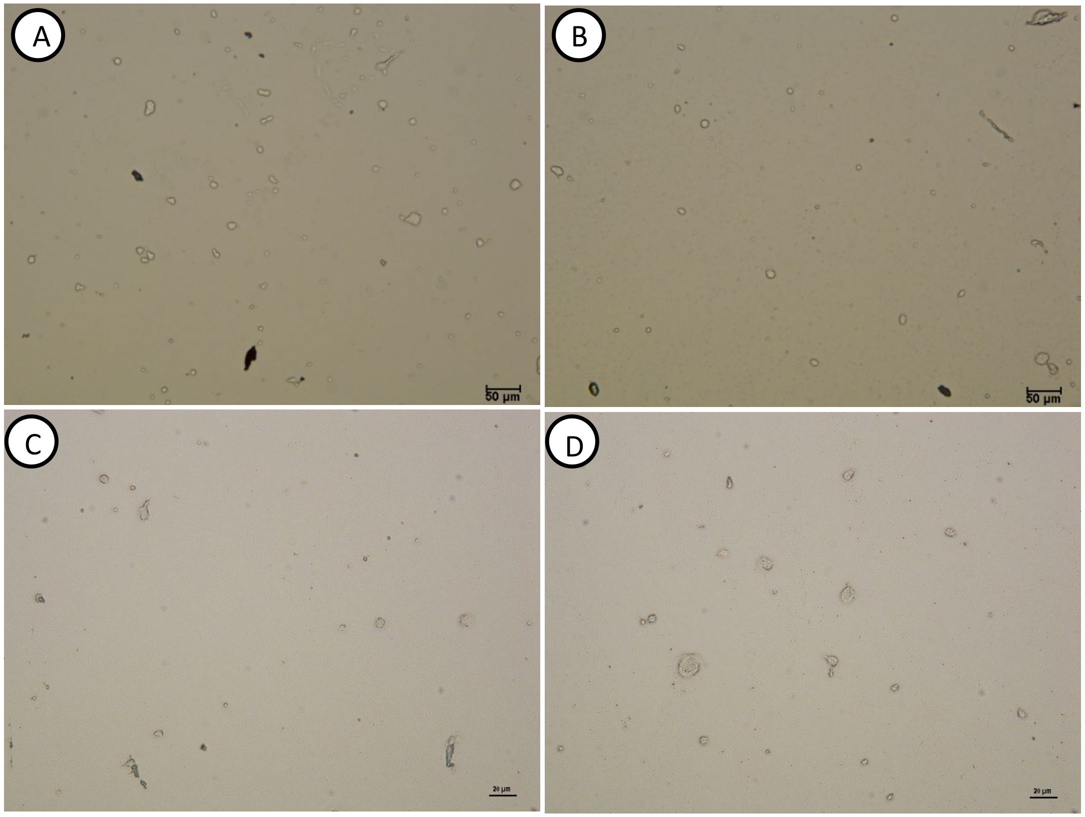

Figure S1. Jicama starch hydrolyzed-based solutions (24 h, 20 % p/v) without voltage (a, b) and after electrospraying (c, d).
